# Predicting Stress Intensity Factor for Aluminum 6062 T6 Material in L-Shaped Lower Control Arm (LCA) Design Using Extended Finite Element Analysis

**DOI:** 10.3390/ma17010206

**Published:** 2023-12-30

**Authors:** Said El Fakkoussi, Sorin Vlase, Marin Marin, Ouadie Koubaiti, Ahmed Elkhalfi, Hassane Moustabchir

**Affiliations:** 1 Mechanical Engineering Laboratory, Faculty of Sciences and Techniques, P.O. Box 2202 Route Imouzzer, Fes 30000, Morocco; ahmed.elkhalfi@usmba.ac.ma; 2 Department of Mechanical Engineering, Faculty of Mechanical Engineering, Transylvania University of Brasov, B-dul Eroilor 29, 500036 Brasov, Romania; 3 Romanian Academy of Technical Sciences, B-dul Dacia 26, 030167 Bucharest, Romania; 4 Department of Mathematics and Computer Science, Transilvania University of Brasov, 500036 Brasov, Romania; m.marin@unitbv.ro; 5 Academy of Romanian Scientists, Ilfov Street, No. 3, 050045 Bucharest, Romania; 6 MSISI Laboratory, Faculty of Sciences and Technics of Errachidia, Moulay Ismail University of Meknes, Meknes 50050, Morocco; o.koubaiti@umi.ac.ma; 7 Laboratory of Systems Engineering and Applications (LISA), National School of Applied Sciences of Fez, Sidi Mohamed Ben Abdellah University, Fez 30000, Morocco; hassane.moustabchir@usmba.ac.ma

**Keywords:** crack, aluminum 6062 T6, lower control arm, stress intensity factor, XFEM, Abaqus/CAE

## Abstract

The aim of this study is to solve a practical problem encountered in the automotive industry, especially the failure of a cracked lower control arm made of al 6062 T6 material during static and crash physical tests, and to characterize the behavior of cracked parts made of aluminum materials using the fracture mechanics parameters. As a first step, we carried out a numerical study and simulation using Abaqus/CAE 2020 software and the finite element method to determine the stress concentration and load limit capacity for different car weight cases. The von Mises stress variation shows crack initiation and propagation to be in the area of the lower control arm’s attachment to the vehicle platform, where stress is concentrated. These numerical results are consistent with the experimental test results found by automotive manufacturers. Also, we find that the mechanical load that can support this part is below 4900 N for good performance. In the second step, we use the results of the first section to simulate the failure of a lower control arm with a crack defect. This paper investigates the stress intensity factor KI in mode I for different lengths (*L*) and depths (*a*) of the crack in the lower control arm using the extended finite element method (XFEM) under Abaqus/CAE. For crack failure initiation and progression, we relied on the traction separation law, specifically the maximum principal stress (MAXPS) criterion. The KI factor was evaluated for the materials steel and Al 6062 T6. The results obtained from the variation of the KI coefficient as a function of crack depth (*a*) and the thickness (*t*) show that the crack remains stable even when a depth ratio (*a*/*t* = 0.8) is reached for the steel material. However, the crack in the Aluminum 6062 T6 material becomes unstable at depth (*a*/*t* = 0.6), with a high risk of total failure of the lower control arm.

## 1. Introduction

The failure of cracked mechanical parts remains a major issue and concern for researchers in the field of fracture mechanics, which is characterized by the complexity of research in a discontinuous domain, and which is always raising concerns about numerical convergence and correlation. In addition, researchers found another complicity in identifying the initiation and path of crack propagation which allows us to anticipate total rupture and call for corrective maintenance to avoid significant material and human damage.

In the automotive industry, the lower control arm is a crucial component for the overall functionality of a vehicle. It plays an important role in stabilizing the vehicle by connecting the vehicle frame to the suspension system. Additionally, it absorbs road noise and ensures optimal comfort for driver and passengers. During operation, they are subjected to several mechanical loads in static and dynamic situations, which create a high risk of initiating a crack or complete failure. In automotive approval, this part has been subjected to several research and validation purposes in different areas such as statics [1,2,3,4], fatigue [3,4,5], dynamics [6], vibration [7], and thermal scenarios [5]. Finally, after these partial validations, the lower control arm will go through another final validation with vehicle synthesis tests for crash tests.

**Figure 1:**
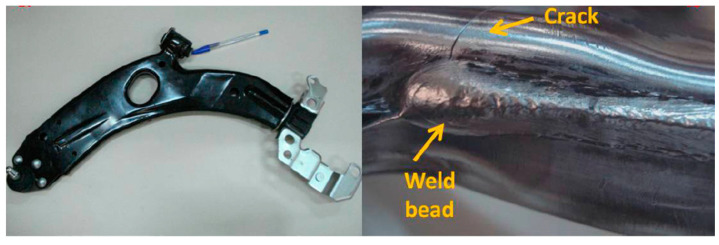
Crack in the lower control arm after fatigue test [9].

Looking at the literature, there are several researchers interested in studying lower control arms in various fields. For example, Abu Bakar et al. [8] tried to predict the weakness areas of the lower control arm via a fatigue analysis using the stress life method. This technique was evaluated using the finite element method with the aim of identifying areas of mechanical weakness and improving the behavior and mechanical performance of this component. On the other hand, Godefroid et al. [9] investigated the root causes of lower control arm failure using fatigue studies (Figure 1) and completed the technical specifications required for proper operation of this system. This study was carried out partly numerically via the finite element method, and the other part is carried out via an experimental study. According to the analysis of the results obtained, the authors found that most cracks started in the welding zone where the stress is concentrated due to the presence of microcracks. Their proposed solution was to develop and modify the existing welding process. Sadiq et al. [10] performed a complete study, including design, static, vibration, and fatigue studies. These different studies were based on numerical simulation via the finite element method, the objective of which was to identify regions of stress concentration in order to be able to determine the zones where they would focus their research for saving mass studies. Yokoyama et al. [11] used the IGA analysis, which is based on the NURS functions, to study the lower control arm in the explicit dynamic domain. This study aimed to integrate the IGA analysis into the numerical crash study, which is fewer errors compared to MEF and XFEM methods.

**Figure 2:**
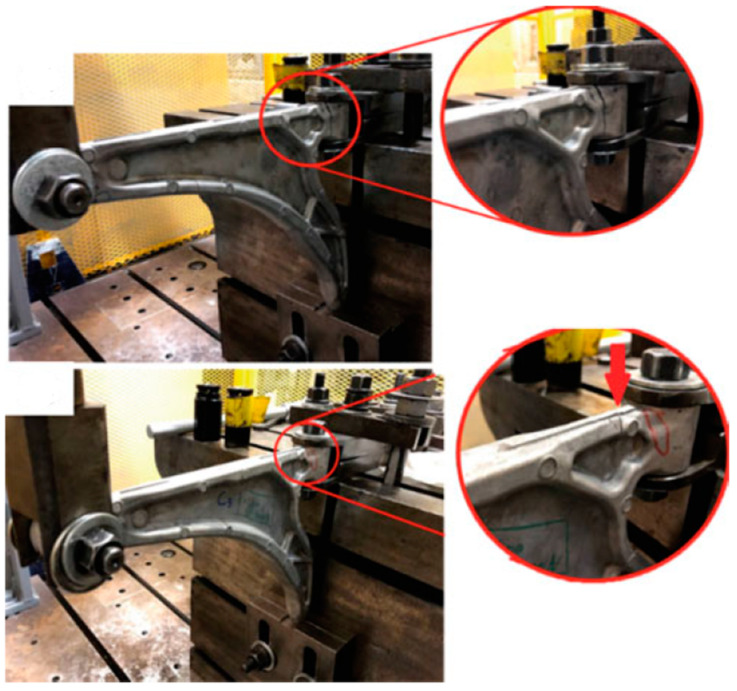
Example of a crack detected during a servo hydraulic fatigue machine test [5].

The lower control arm is usually made of steel in thermal vehicles, as it offers a high price/performance ratio. However, with the advent of new car electrification technology, car manufacturers are aiming to reduce weight to enhance the performance and autonomy of electric vehicles. According to this, several researchers have tried to further develop optimization tools which make it possible to find a good weight range and maintain good performance, including composite materials for good performance and weight, but the disadvantage of these materials is their high price. We found that the aluminum material has a good weight/performance ratio and price. Evaluating the lower control arm with aluminum material 6062 T6, known for its mechanical strength, light weight, corrosion resistance, and feasible manufacturing process, becomes a pertinent consideration. Several researchers, including Jiang et al. [12], Zhao et al. [13], and Messana et al. [7], have carried out mass optimization studies in the vibration field. They conducted the study in two parts: a numerical part and an experimental part. In this sense, Mohamed Attia et al. [5] have carried out a fatigue study of the lower control arm in aluminum material A357 in a dynamic case (Figure 2), and they pushed further the study of heat treatment in order to attain the mechanical and thermal properties of the lower control arm in aluminum material compared to steel materials.

During the manufacturing process, some parts may develop defects that are not detected during quality control. These defects pose a major challenge to car manufacturers as they can potentially affect performance, leading to overall damage and even accidents. In response to this issue, we propose to study the lower control arm with a pre-crack and aim to predict its impact on the performance of the system wishbone. To carry out this research, we based our objective of studying the mechanical behavior of a structure in the presence of a macroscopic cracks and the adjacent continuous medium, as well as the propagation of this discontinuity over time in metal structures, on fracture mechanics. This amounts, in particular, to determining the field of stress and deformation in the vicinity of the crack tip [14]. The literature implements certain methods based on the concept of stress intensity factor (KI) by making it possible to precisely evaluate the rupture of cracked structures. This KI factor is determined analytically or via numerical methods. To calculate the latter, we will use the extended finite element method and its capabilities in the modeling and calculation of mechanical parameters at failure in the discontinuous domain (the presence of a crack).

In the literature, Kishorekanna et al. [15] conducted a performance analysis of a U-shaped pre-cracked lower control arm by applying the finite element method. This study integrated the concepts of fracture mechanics to evaluate the stress intensity factor (KI) in the stress concentration zone of the lower control arm. The fracture mechanics are useful for estimating the structural responses of components caused by microcracks in the stress concentration zone.

In a similar context, Saurabh Gairola et al. [16] conducted both numerical and experimental studies to evaluate the KI factor in several specimens of aluminum material. Additionally, Fageehi [17] studied the initiation and propagation of the crack under the effect of mixed loading on a 3D specimen made of aluminum 7075 T6 and SAE 1020 steel materials. He based his study on the law of Paris to assess the influence of a hole on the propagation of the crack. In the case of a fatigue studies, he also calculated the stress intensity factor (KI) and predicted the direction in which the crack will propagate using the extended finite element method (XFEM).

A literature review conducted to highlight the uniqueness of our study concludes that the geometry we are about to study has not been studied. Research into the fracture of the lower control arm in the presence of cracks has been little or rarely studied. The crack treatment for this type of geometry using the extended finite element method (XFEM) has not been carried out. Here, we characterize the behavior of cracked parts made of aluminum materials using the fracture mechanics parameters.

The objective of this study is to propose numerical analysis using Abaqus/CAE software and the finite element method to determine the stress concentration and load limit capacity for different car weight cases. We use fracture mechanics field to study the stress intensity factor (KI) in mode I for different lengths (*L*) and depths (*a*) of the crack in the lower control arm, using the extended finite element method (XFEM) under Abaqus/CAE. The KI factor was evaluated for the materials steel and Al 6062 T6. This study aims to provide additional analysis of the static case and complement previous studies conducted primarily in dynamic, fatigue, and vibration scenarios. Additionally, we aim to provide input data for future optimization studies, with a special focus on the vulnerabilities that need to be carefully considered during analysis.

## 2. Theoretical Background

### 2.1. Overview of Extended Finite Element Method

The XFEM method is used to treat cracked structures. The formulation of this method consists of two parts: the first part is the classical finite element method, which manages the continuous area of the structure; and the second part is used to evaluate the crack. The evaluation of the crack zone is performed in two parts: the first part deals with the stress singularity at the crack tip; and the second part aims to model the crack jump using the Heaviside function *H*(*ξ*). The advantage of this method is that it is capable of modeling the initiation and propagation of cracks without the need for a specific mesh like the classic method, which saves computational time and good convergence.

The displacement formula, according to the extended finite element method (XFEM) written by researchers Belytschko and Moës [18], is expressed as follows:

(1) $$u\left(\xi \right)={u\left(\xi \right)}^{FEM}+{u\left(\xi \right)}^{Enrichment}$$

(2) $$u\left(\xi \right)=\sum_{i}^{n}N_{i}\left(\xi \right)u_{i}+\sum_{j}^{n_{cf}}N_{j}\left(\xi \right)\left[H\left(\xi \right)-H\left(\xi_{j}\right)\right]a_{j}+\sum_{k}^{n_{ct}}N_{k}\left(\xi \right)\sum_{\alpha =1}^{4}\left[\Psi_{\alpha }\left(\xi \right)-\Psi_{\alpha }\left(\xi_{k}\right)\right]b_{\alpha k},$$

where:

ξ=(x,y): coordinates of the global reference;
$N_{i}\left(\xi \right)$: the classical finite element interpolation function;
$H\left(\xi \right)$: the Heaviside function enrichment represented the crack edge;
$a_{j}$: the additional degrees of freedom related to the Heaviside function;
$\Psi_{\alpha }\left(\xi \right)$: the enrichment function which models the stress singularity in the vicinity of the crack tip;
$b_{\alpha k}$: the additional degrees of freedom associated with singular functions.

A necessary condition to ensure convergence is that the shape functions $N_{i}\left(\xi \right)$, $N_{j}\left(\xi \right)$, and $N_{k}\left(\xi \right)$ create a device partition of the domain. The function $H\left(\xi \right)$ is presented by the following equation:

(3) $$H\left(\xi \right)=\left\{\begin{array}{c} +1\;\;\;\varphi (\xi )>0\\ \;\;\;\;0\;\;\;\varphi \left(\xi \right)=0\\ -1\;\;\;\;\varphi (\xi )<0 \end{array}\right..$$

**Figure 3:**
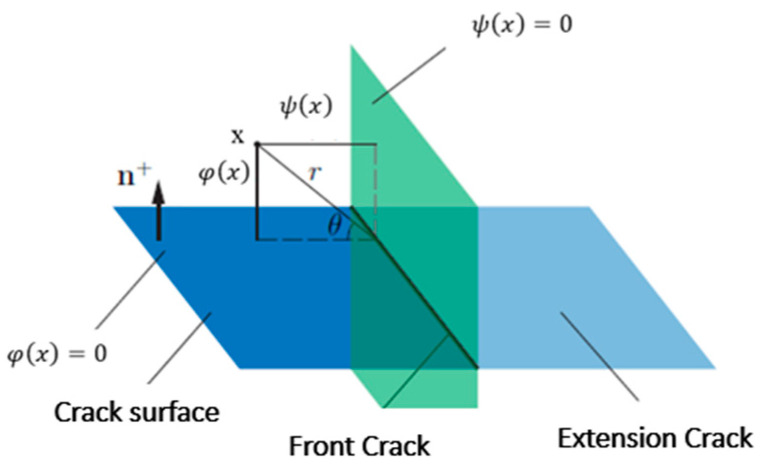
Level set method approach to represent the geometry of the crack.

The function $H\left(\xi \right)$, therefore, takes the values (+1) or (−1) depending on the side of the crack on which it is placed. A crack is described by two sets of levels: a normal level-set $\varphi \left(\xi \right)$ is the function which gives the distance from a point *ξ* to the surface of the crack (Figure 3); a tangential level-set $\psi \left(\xi \right)$ is the function which gives the tangential distance from a point *ξ* to the bottom of the crack (Figure 3).

**Figure 4:**
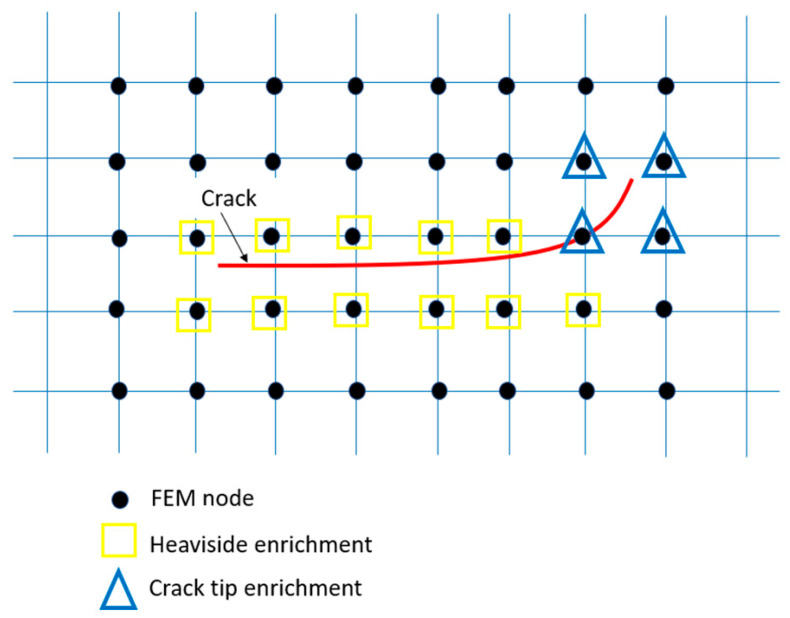
Strategy of enrichment of a crack via the XFEM method.

The enrichment function $\Psi_{\alpha }\left(\xi \right)$ refers to the singularity in the vicinity of the crack tip (Figure 4). It is represented by the following equation:

(4) $$\Psi_{j}\left(r,\theta \right)=\left\{\sqrt{r}\sin\left(\theta /2\right),\sqrt{r}\cos\left(\theta /2\right),\sqrt{r}\sin\left(\theta /2\right)\sin\left(\theta \right),\sqrt{r}\cos\left(\theta /2\right)\sin\left(\theta \right)\right\}$$

(5) $$\begin{array}{c} r=\sqrt{\varphi^{2}\left(\xi \right)+\Psi^{2}(\xi )}\\ \theta =\;\;\;\;\;\;arctan\left(\frac{\varphi (\xi )}{\Psi (\xi )}\right) \end{array},$$

or,

$\left(r,\;\theta \right)$ is the polar coordinate system and its origin at the crack tip.

An important further development of XFEM was made regarding its combination with the “level set” feature [19]. The level set function provides information about where the singularity is and how to strengthen it. The functions of the level set method (LSM) [19] are interpolated by the functions of Lagrange interpolation:

(6) $$\left\{\begin{array}{c} \varphi \left(\xi \right)=\sum_{i=1}^{n}N_{i}(\xi )\varphi_{i}\\ \Psi \left(\xi \right)=\sum_{i=1}^{n}N_{i}(\xi )\Psi_{i} \end{array}\right..$$

### 2.2. Stress Intensity Factor KI

Fracture mechanics offers several parameters with which to identify the initiation and propagation of the crack; among these parameters we find the stress intensity factor (KI), which can predict the initiation and propagation of a crack in comparison with material toughness KIC. This critical coefficient depends on the mechanical properties of the material; it is calculated experimentally using the Charpy V test. Several methods for calculating stress intensity (KI) are found in the literature, either analytically or numerically, based on a displacement extrapolation or based on an energy approach; for example, the J integral or the interaction integral. In the computational code Abaqus/CAE, the evaluation of integral J is automated using the method of the integral of the energy domain [20]. This method is based on the formulation provided in Equation (7):

(7) $$J=\int_{A^{*}}\left[\sigma_{ij}\frac{\partial u_{j}}{\partial x}-w\delta_{1i}\right]\frac{\partial q}{\partial x_{i}}dA,$$

where A* is the area of the surface between the contours $\Gamma_{0}$ and $\Gamma_{1}$. The parameter (*q*) is the smoothed selection function; *q* = 1 on $\Gamma_{0}$ and *q* = 0 on $\Gamma_{1}$. The contour $\Gamma_{1}$ is reduced in practice at the crack tip. The outline $\Gamma_{1}$ coincides with the edges of the elements.

The calculation of the KI factor in the mechanical fracture is also based on the energy approach, especially the *J* integral. It was first proposed by Rice in 1968 [21]; in the case of a single mode for the problems linear elasticity, the integral *J* is equal to energy restitution G. The factor KI in mode I (opening case) is calculated as a function of the integral *J* as presented by the following equation:

(8) $$K=\sqrt{JE^{\prime }},$$

where $E^{\prime }=E$ plane stress; $E^{\prime }=\frac{E}{1-\nu^{2}}$ plane strain; *E* = Young’s modulus; and ν = Poisson’s ratio.

Elsewhere, the stress intensity factor ($K_{I}$) is written as follows:

(9) $$K_{I}=Y\sigma \sqrt{\pi a}.$$

with:

a: the size of the crack;
σ: the constraint applied, depending on the type of loading and the test specimen used;
*Y*: geometric factor, depending on the geometry and size of the crack (*a*).

Estimating the stress at the crack tip, and in particular the stress intensity factor *K_I_*, allows us to predict the initiation of propagation. The energetic approach makes it possible to study both the initiation of propagation and its control. For mode *I*, there is the initiation of the crack when the parameter KI reaches a critical value *K_IC_* called toughness fracture of the material [21]. This toughness makes it possible to characterize the resistance to sudden propagation of the crack.

(10) $$\begin{array}{c} K_{I}<K_{IC}\;\;\;\;\;\;\;\;\;\;\;non\;propagation\\ K_{I}={\;K}_{IC}\;propagation\;\;\;\;\;\;\;stable\\ K_{I}>{\;K}_{IC}\;\;propagation\;\;\;\;instable \end{array}$$

## 3. Computational Modelling

### 3.1. Design

**Figure 5:**
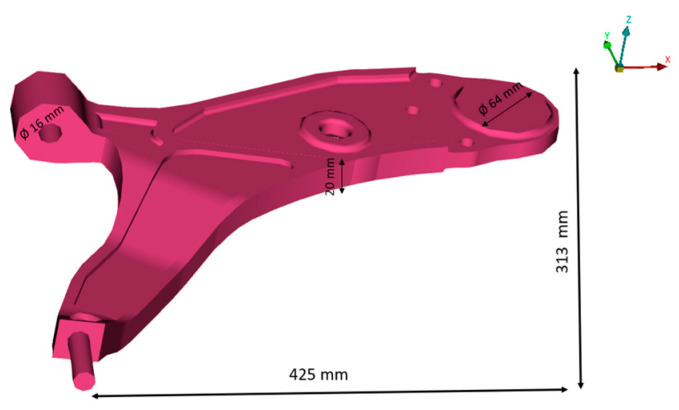
Dimensions of the lower control arm and final CAD model.

The geometry that we will study is characterized by thickness (*t*), width, and length, as detailed in Table 1 and Figure 5 below.

**Figure 6:**
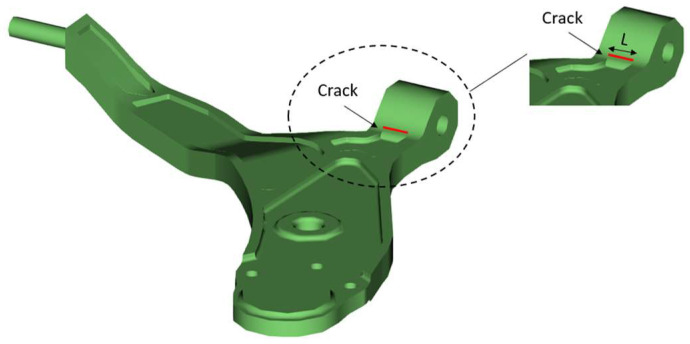
Dimensions the default in the lower control arm.

According to reference [9,13,22,23], all the defects detected during fatigue testing or during operation of the lower control arm appear at the end of the part show in Figure 6. To complete these studies by these cited researchers, we will propose that the defect resulting from the manufacturing process be reproduced in the same area. This crack is characterized by several study values of a length *L* (*L* = 10 mm and *L* = 20 mm) and a depth *a* (*a* = 5 mm, *a* = 8 mm, *a* = 10 mm, *a* = 12 mm, and *a* = 16 mm).

### 3.2. Material

The mechanical characteristics of the material used in our is presented in Table 2.

### 3.3. Meshing, Loadings, and Boundary Condition

**Figure 7:**
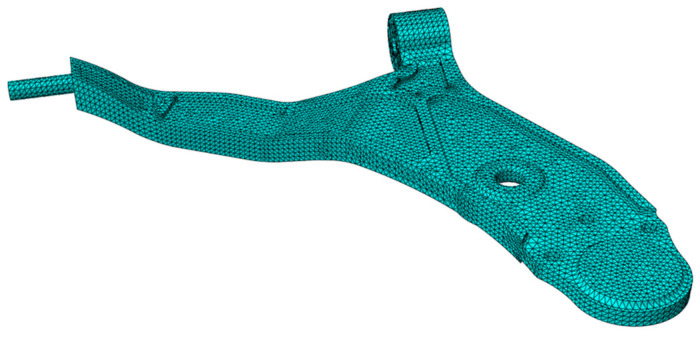
The mesh of the lower control arm, 3D Stress C3D20R (quadratic), with 4.4 mm of size; 130,778: total number of nodes; 84,839: total number of elements.

The step of meshing the geometry studied, which conditions the accuracy of the results and calculation time, is an important one. In the XFEM method, the mesh geometry is independent of the crack. Because the enrichment functions at the edge of the crack take a role in modeling the discontinuous region, it is the power of this method compared to the classical finite element method which requires a specific mesh at the crack tip (Figure 7).

**Figure 8:**
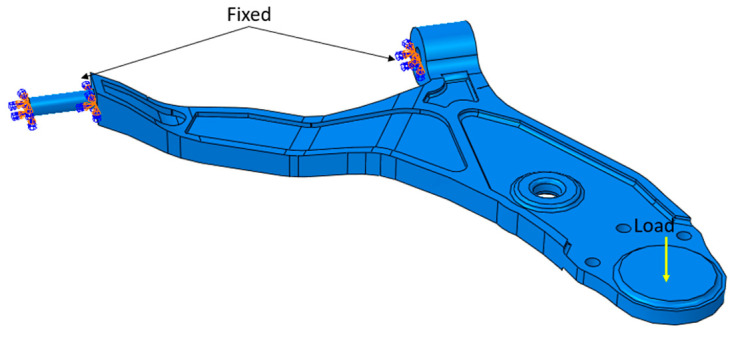
Lower control arm boundary condition.

For the loading part, we will apply several mechanical loads to the lower control arm, as shown in Figure 8. Each loading models the weight of the front axle of the car that can support the lower control arm. With this assessment, we will be able to determine the limits that are supportable by the lower control arm and to determine the technical specifications of this part. The applied loads are equivalent to the weight of the car when stationary (1600 N, 4900 N, 5500 N, and 7000 N).

### 3.4. The Extended Finite Element Analysis

To model the initial crack and crack propagation in Abaqus/CAE using the XFEM method, it is necessary to adopt the linear traction–separation law. This law is characterized by three parameters: cohesion force, cohesion energy, and critical displacement [15]. The evolution of the fracture is modeled according to three specific stress-based criteria: maximum principal stress (MAXPS), maximum nominal stress (MAXS), quadratic nominal stress (QUADS). It is also modeled on three criteria specifically based on strain: maximum principal strain (MAXPE), maximum nominal strain (MAXE), and quadratic nominal strain (QUADE). In the literature [15,20], we find that several studies are based on the MAXPS criterion to model the initiation and propagation of the crack. In our study, we based our analysis on the maximum nominal stress (MAXPS) and critical displacement.

## 4. Results and Discussion

This work is organized into two parts. In the first part, we study the lower control arm in the elastic domain without the defects to characterize this part and determine the maximum load it can support. In the second part, we assume the presence of a defect in the form of a crack in the critical region detected in the first part; then, we use the XFEM method to extract the KI coefficient of the cracked lower control arm and compare it with the fracture toughness KIC.

**Figure 9:**
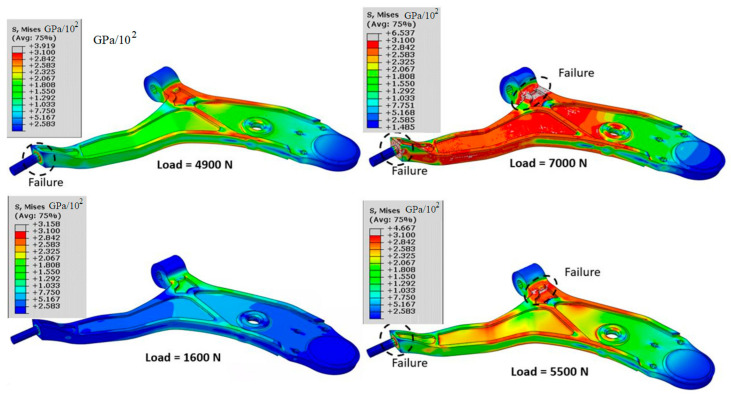
Von Mises stress distribution for different loads.

Figure 9 below shows the stress distribution for different loads (1600 N, 4900 N, 5500 N and 7000 N); these loads represent the weight of the car’s front axle that can support the lower control arm in the static case when the vehicle is stationary.

The obtained results show that there are two weak points in this part, especially in the part attached to the frame vehicle. This failure can be seen when applying loads of 5500 N and 7000 N. With these loads, we exceed the ultimate tensile strength; thus, the lower control arm fails with these loads (Table 3). On the other hand, we find two favorable cases when we apply loads of 1600 N and 4900 N: for the first case, the results are below the breaking limit; and the same is observed for the second load when we are near the limit of rupture, but it remains acceptable. From these results, we conclude that the load or weight that can support this part is less than 4900 N.

According to the results of the first part, we will use the loads of 1600 N and 4900 N to study the lower control arm, but this time with the presence of a crack defect with length *L* and depth *a*.

**Figure 10:**
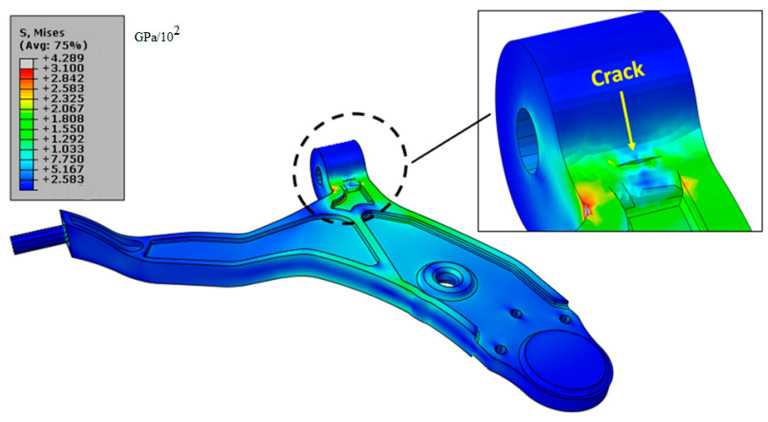
von Mises stress distribution with the presence of a crack depth (*a*) and thickness (*t*) *a*/*t* = 0.25 of length *L* = 10 mm, with a loading of 1600 N.

Figure 10 shows the von Mises stress distribution near a crack with a length of 10 mm according to the ratio crack depth (*a*) and the thickness (*t*) (*a*/*t* = 0.25). The stress is concentrated at the ends of the crack; also with the presence of this crack, the stress found is a little close to the limit of the material failure. From these results, it can be concluded that this crack presents small risk to the lower control arm in the static case. And in the second section, we will quantify the risk using the parameters of fracture mechanics, in particular the KI factor.

**Figure 11:**
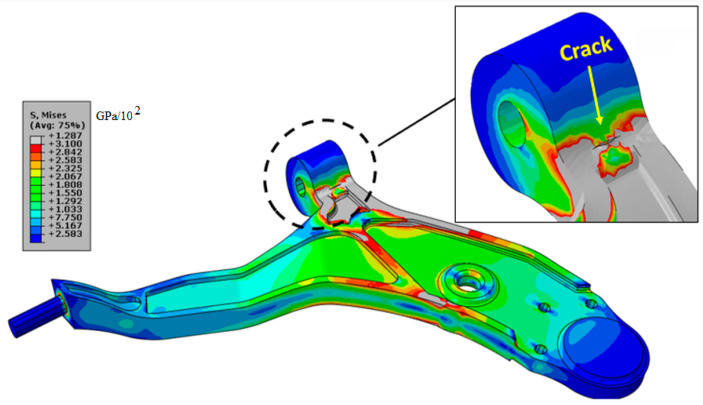
von Mises stress distribution with the presence of a crack *a*/*t* = 0.25 of length *L* = 10 mm with a loading of 4900 N.

On the other hand, we evaluated the same crack size but this time with a higher load, equivalent to 4900 N. The obtained results are presented in Figure 11. It can be seen that in some areas, the von Mises stress exceeds the rupture limit of the ultimate tensile strength material. We conclude that this load, in the presence of a crack of length 10 mm, presents a very high risk of rupture for a weight of the front axle of the car equivalent to a load of 4900 N.

**Figure 12:**
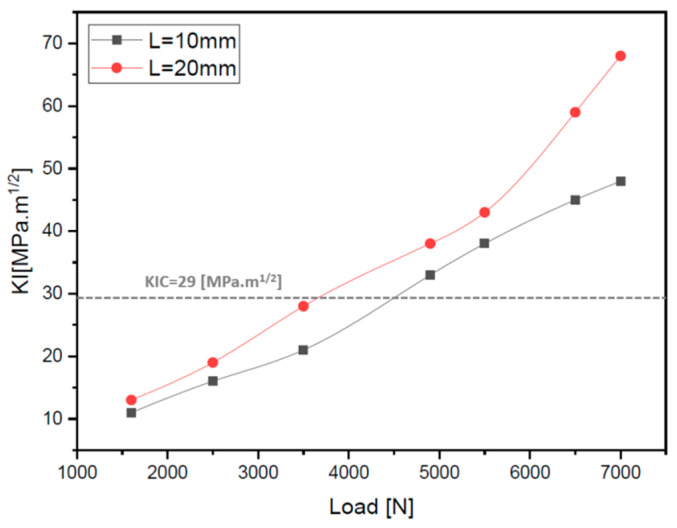
Variation of the KI factor depending on the increase in loads for a crack depth (*a*) and the thickness (*t*) *a*/*t* = 0.25.

For further the evaluation of the risk of failure with different crack sizes and for different loads, we based our study on the stress intensity factor KI using the XFEM method via the Abaqus/CAE software. The calculation results of this factor are considered from three contours for good accuracy and estimation. We will use the critical stress intensity factor KIC, which depends on the material, to deduce whether the crack is stable or not. Figure 12 shows the variation of the KI factor as a function of load. The results obtained show that when the load of 4000 N is exceeded, the crack propagates towards the end of the part and thus leads to total fracture. We also conclude that the risk increases as the crack becomes wider, as shown by the red curve in Figure 12. These results are consistent with those presented in Section 1, which are based on the analysis of the stress around the crack.

**Figure 13:**
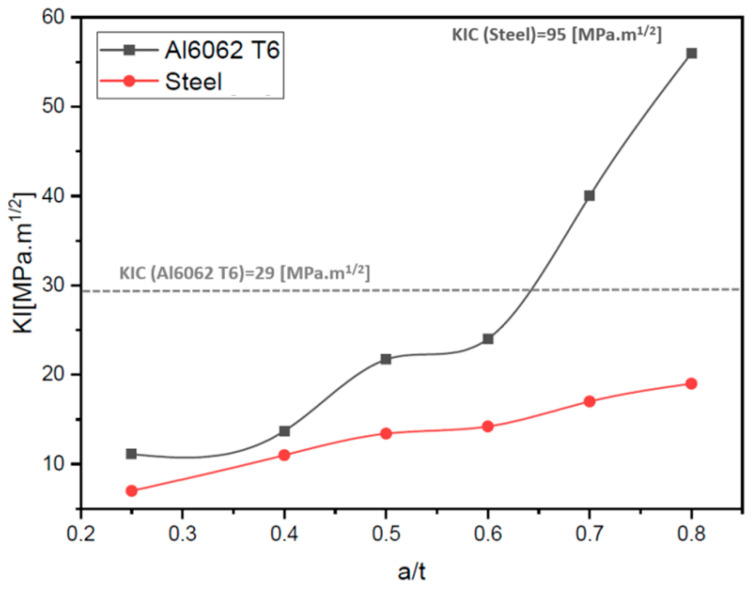
Variation of the KI factor depending on the depth of the crack for a loading of 1600 N [22].

To complete the results, we will try to evaluate the influence of the crack depth on the calculation of the KI factor. To do this, we will take a 1600 N load case with a crack length of 10 mm, the objective of which is to determine the impact on the performance of the lower control arm. Figure 13 shows a comparison between steel materiel and 6062 T6 aluminum materiel to calculate KI based on crack depth. The results obtained show that with the steel material, the crack is stable even if we arrive at a depth ratio crack depth (*a*) and the thickness (*t*) (*a*/*t* = 0.8). However, we found that the crack in the aluminum 6062 T6 material from a depth of 0.6 was no longer stable and led to a very high risk of total failure of the lower control arm.

From the results, we concluded that if we reassemble a cracked lower arm control, which was not detected during quality control on a vehicle, the lower control arm remains functional and would not present any risk if the defect ratio remained less than 60%. On the other hand, on steel material, we can reach a depth of 80%. The results of this work provide the technical specifications required for the operation of this type of lower control geometry in cases without or with the presence of a defect for steel and aluminum materials. Also, these results remain useful as input data for automobile manufacturers in future performance improvement studies and mass optimization studies undertaken in order to find a good compromise between performance and manufacturing cost price.

## 5. Conclusions

The lower control arm is an important part in the vehicle’s operation; it connects the vehicle frame and the suspension system. According to the numerical analysis in the static case, we find that the stress concentration is located in the areas where the lower control arm is attached to the vehicle frame. The results show that with the Al 6062 T6 material, the lower control arm supports a force of up to 4900 N.

On the other hand, we analyzed the presence of cracks due to manufacturing defects within the stress concentration area, as above. We used the extended finite element method (XFEM) to treat the cracked lower control arm because this method allows us to calculate the KI factor with good accuracy, and it allows us to determine whether the fracture is stable compared to the KIC. Fracture toughness depends on the material.

We conclude from the results obtained that as the crack becomes wider in the lower control arm made of Al 6062 T6 materiel, the risk of crack propagation increases; on the other hand, we find that the crack is stable on the steel material. We also concluded that the lower control arm in Al 6062 T6 remains functional in the presence of cracks with a ratio (*a*/*t*) less than 0.5 and with load less than 1600 N.

For perceptive purposes, the results of this work provide technical specifications for lower arm control in aluminum and steel with respect to proper operation in the presence of a defect due to the undetected manufacturing process. In addition, these results remain usable to prepare the preventive maintenance plan for the wishbone system. Moreover, these results serve as input data for future mass optimization studies for good performance in order to maintain good car safety, protecting the driver and passengers.

The next research project is to add a complementary study in the thermal and dynamic cases in the presence of a crack on the lower control arm in order to evaluate the mechanical behavior and performance of the lower control arm.

## Figures and Tables

**Table 1:** Dimensions of the lower control arm.

Thickness (*t*)	Fixation Diameter	Width	Length (*L*′)
20 mm	16 mm	425 mm	313 mm

**Table 2:** Mechanical properties of AL6062 T6.

Material	Elastic Modulus (GPa)	Poisson’s Ratio	Yield Strength (MPa)	Ultimate Tensile Strength (MPa)	Elongation at Break	Fatigue Resistance (MPa)	Fracture Toughness KIC (MPa·m^1/2^)
6062 T6	68.9	0.33	276	310	12%	96.5	29

**Table 3:** Maximum stress values for different loads.

Von Mises Stress (MPa)	315	391	466	653
Load (N)	1600	4900	5500	7000

## Data Availability

Data are contained within the article.
